# SPEACH_AF: Sampling protein ensembles and conformational heterogeneity with Alphafold2

**DOI:** 10.1371/journal.pcbi.1010483

**Published:** 2022-08-22

**Authors:** Richard A. Stein, Hassane S. Mchaourab

**Affiliations:** Department of Molecular Physiology and Biophysics, Vanderbilt University, Nashville, Tennessee, United States of America; University of Oxford, UNITED KINGDOM

## Abstract

The unprecedented performance of Deepmind’s Alphafold2 in predicting protein structure in CASP XIV and the creation of a database of structures for multiple proteomes and protein sequence repositories is reshaping structural biology. However, because this database returns a single structure, it brought into question Alphafold’s ability to capture the intrinsic conformational flexibility of proteins. Here we present a general approach to drive Alphafold2 to model alternate protein conformations through simple manipulation of the multiple sequence alignment via *in silico* mutagenesis. The approach is grounded in the hypothesis that the multiple sequence alignment must also encode for protein structural heterogeneity, thus its rational manipulation will enable Alphafold2 to sample alternate conformations. A systematic modeling pipeline is benchmarked against canonical examples of protein conformational flexibility and applied to interrogate the conformational landscape of membrane proteins. This work broadens the applicability of Alphafold2 by generating multiple protein conformations to be tested biologically, biochemically, biophysically, and for use in structure-based drug design.

## Author summary

Many questions have arisen with the remarkable protein prediction capability of Alphafold2. One of the main questions is whether Alphafold2’s architecture is amenable to reveal the intrinsic conformational heterogeneity of proteins. The potential to obtain unseen or hidden conformations with high accuracy would greatly advance a broad range of structural biology pursuits. We have devised a method of *in silico* mutagenesis of the multiple sequence alignments (MSA) that are central to Alphafold2’s prediction capabilities. The approach consistently unveils conformations not seen with the unmodified default MSA. Analysis of the ensembles of predicted conformations relative to experimental structures fully support the biochemical significance of the models.

## Introduction

The explosion of complete sequencing for a multitude of genomes has allowed for the generation of deeper multiple sequence alignments (MSA). These MSAs are a treasure trove of information encoding co-evolution of residues that may be far apart in the linear amino acid sequence. Multiple groups have harnessed co-evolution of residues to generate distance restraints/matrices and the subsequent construction of a three-dimensional protein structure [1,2]. The latest iteration, Alphafold2 (AF2), took a significant leap forward with the quality of its predicted structures [3,4].

A database of structural models for multiple proteomes generated by AF2 has been released (www.alphafold.ebi.ac.uk). The database contains a single conformation for each protein sequence following Anfinsen’s principle that the amino acid sequence determines the native fold of the protein [5]. However, the deposition of a single structure for each protein belies the true ensemble nature of proteins which often undergo functionally important conformational changes. The ensemble nature and conformational heterogeneity of most protein structures would therefore argue that a protein sequence also encodes for structural diversity. The implication is that the distance matrix, which AF2 derives from the MSA, contains information on this heterogeneity although at present, the general consensus is that AF2 is only able to predict a single conformation.

Here, we develop a general approach to transcend this apparent limitation of AF2 and consequently predict ensembles of conformations. Our work was stimulated by the modeling of the Deepmind team of multiple conformations of the multi-drug transporter LmrP (T1024) in their most recent CASP submission, which they achieve by manually curating structural templates [6]. It was noted, based on the MSA and structures for LmrP homologs, that there should be more than one conformation, including an inward- and outward-open conformation. Although the initial runs of AF2 yielded only the inward-open conformation, the MSA derived distance matrix predicted regions that should be close, but were in fact far apart in the computed structure [6]. The submission to CASP XIV entailed paring the MSA and limiting the structural templates to outward-open structures, which yielded the alternate outward-facing conformation of LmrP.

Recently, a methodology for sampling protein conformational space with AF2 was described [7]. To obtain multiple conformations, several alterations in the AF2 pipeline were made: there was no recycling within the AF2 module and the number of sequences in the MSA input was reduced. The authors tested this modified pipeline on a set of 8 protein targets where none of the structures were in AF2’s training set. Remarkably, they were able to obtain both conformations in 7 targets without templates and in the last case with templates. In contrast, this approach failed for 4 targets where one of the structures was present in AF2’s training set, suggesting that in these cases AF2 may default to learned structures.

**Fig 1:**
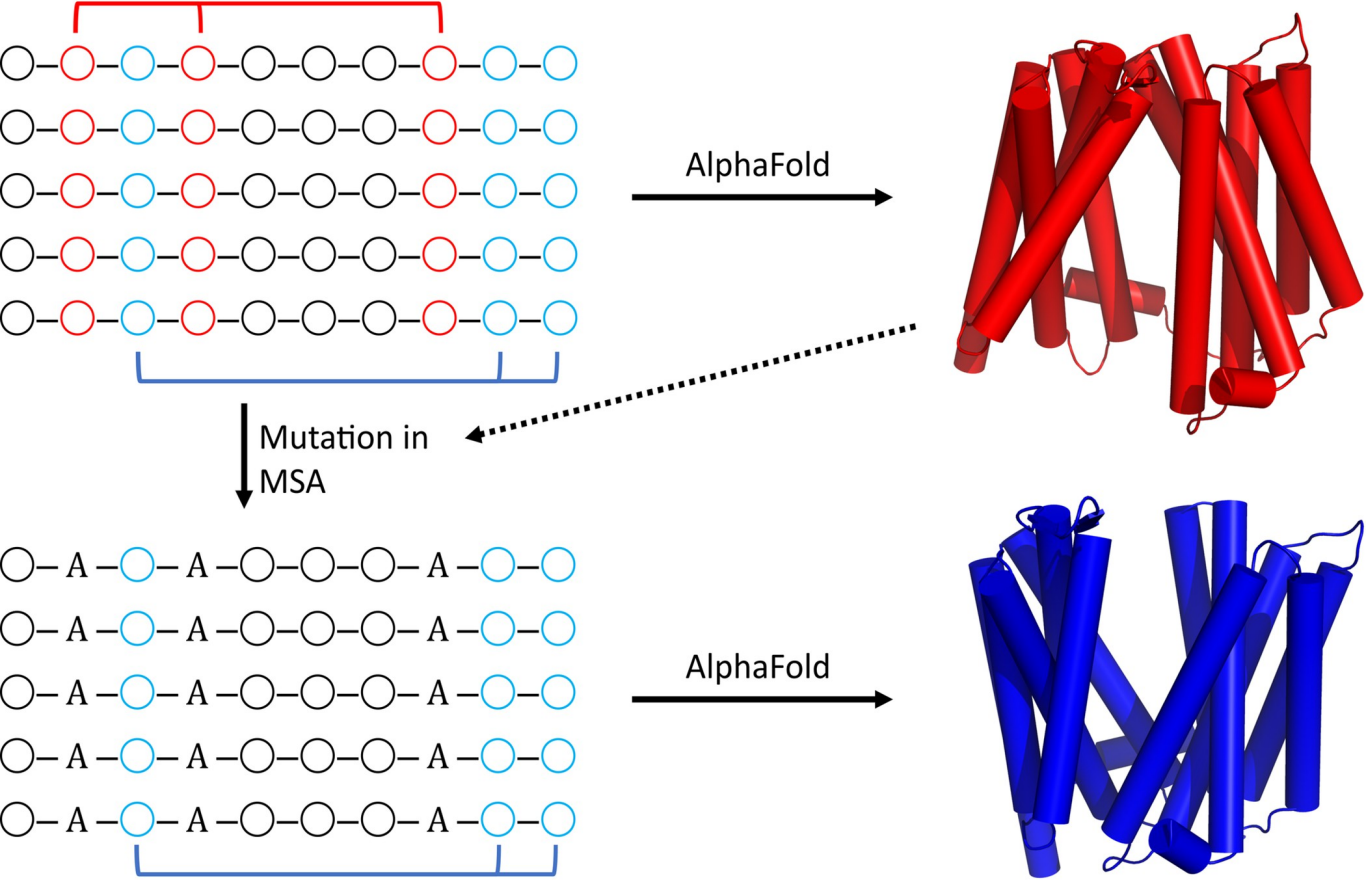
Methodology. An initially generated MSA, via MMSeqs2, is input into Alphafold2 within ColabFold to generate five structural models. For illustration, the model with the highest pLDDT, AF2’s ranking of model confidence, is shown in red. Residues for mutation are chosen, in this case the three residues in red mediating a contact point on the upper surface of the protein. These mutations are made across the entire MSA (ignoring gaps). This modified MSA is then input into ColabFold for generation of new models. With the contact points in red missing, Alphafold2 within ColabFold generates a new conformation based on the contacts shown in blue.

This study introduces an alternative method for biasing the models generated by AF2 that transcends the apparent bias of AF2 to conformations in its training set. The method entails replacing specific residues within the MSA (*in silico* mutagenesis) to potentially manipulate the distance matrices leading to alternate conformations. In outline, AF2 initial models are scanned to identify interaction surfaces within the structure. The MSA is then modified, by *in silico* alanine mutagenesis, either in a systematic manner or based on possible contact points in the initial AF2 structure and/or prior structural information (Fig 1). Within AF2 is an attention network that ascertains the co-evolution of amino acid residues from the MSA. The rationale of our method is that alteration of a set of amino acids to alanine or other residues across the MSA turns the attention of the network to different parts of the MSA allowing for AF2 to find alternative conformations based on other co-evolved residues. Our methodology goes a step further than testing the validity of AF2 models by interrogating the conformational landscape of the protein in the context of the underlying biochemical function.

## Results

Protein targets were selected to illustrate the general applicability of this method and to investigate its limitations. They include two classical examples of protein flexibility, adenylate kinase and ribose binding protein; four membrane proteins where only one conformation was in the AF2 training set; and eight membrane proteins where both conformations were not in the AF2 training set. To enable direct comparison, the twelve membrane proteins were the same as those used in a previous study exploring conformational sampling with AF2 [7]. In summary, the choice of residues to alter in the MSA entails determining sites of interactions within the target using an 11 amino acid sliding window along the best pLDDT (the predicted local-distance difference test which is Alphafold’s metric for ranking the confidence in the structure at every residue) scoring model from an initial AF2 run without templates. The sites of interaction are then modified in the MSA to alanines across all sequences where there is a non-gap residue. This modified MSA alignment is then used 3 times, varying the random seed, to generate 15 models from AF2. The unmodified MSA is also run additional times to obtain 15 initial models.

### Canonical examples of conformational flexibility

#### Adenylate Kinase

**Fig 2:**
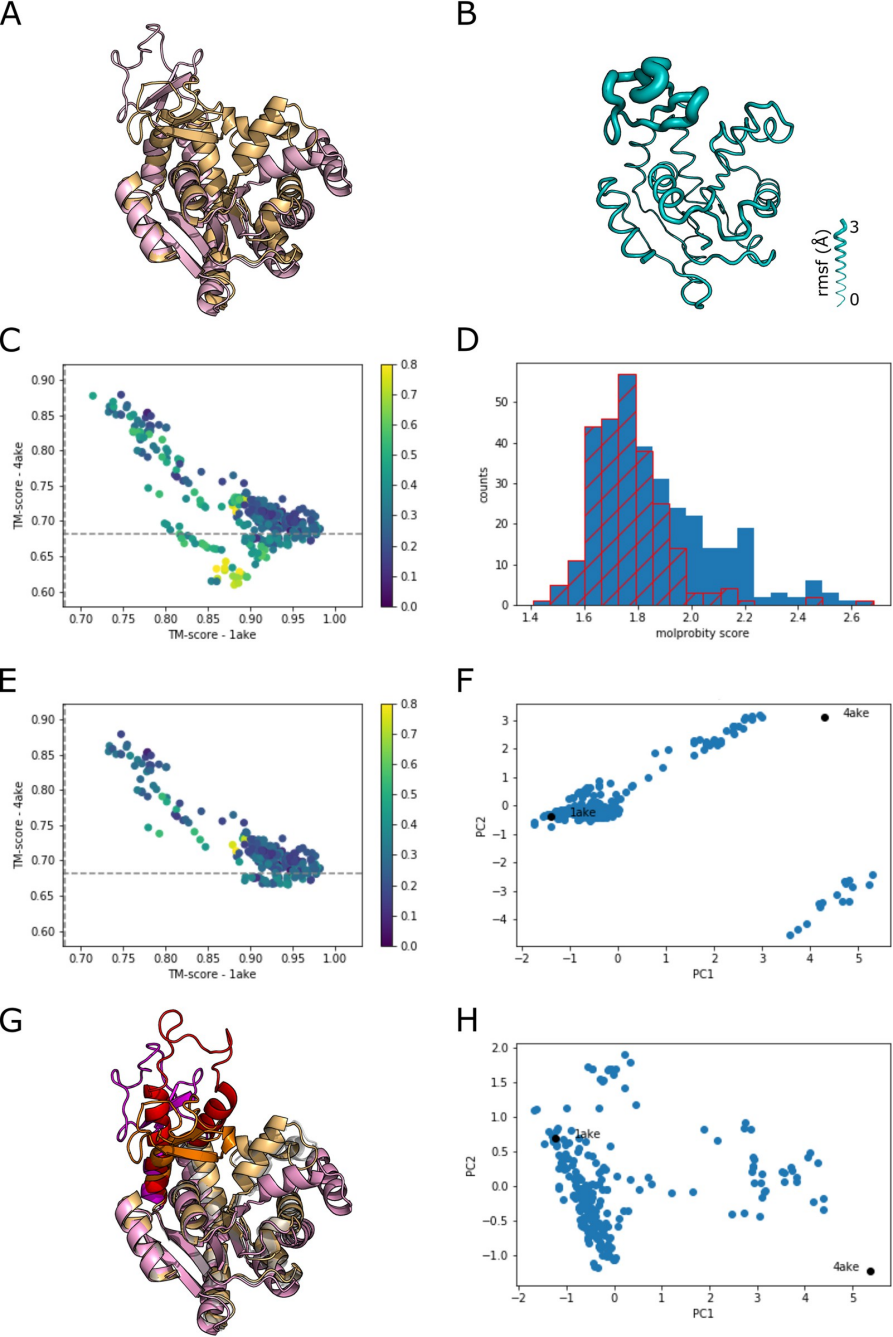
Sampling conformational flexibility of Adenylate Kinase (AK). A) Crystal structures of Adenylate Kinase aligned on residues 1–25: closed, 1ake (lightorange) and open, 4ake (lightpink). B) AK model with the thickness of the chain based on the rmsf for the 15 AF2 models from the unmodified MSA. C) TM score plot comparing all AF2 models to the two crystal structures. The dashed line is the TM score between the two experimental structures. The color scale is based on the relative molprobity score, (MP–MPmin) / MPmin. D) Histogram of molprobity scores. In blue are all of the models. The hatched red plot is for the models parsed by excluding sets that are one standard deviation above the median of all models. E) TM score plot of the parsed set of models. F) PCA plot of the first two components for the parsed set of models. Note the outlier set to the lower right. G) Highlighted is the ATP binding region, 1ake (orange), 4ake (magenta), and a representative structure from the outliers in ‘F’ (red). H) PCA plot removing the misfolded models shown in ‘G’.

Adenylate Kinase (AK), considered a canonical example of protein flexibility, is a nucleoside monophosphate kinase that catalyzes the reaction ATP+AMP ⇌ 2ADP. Crystal structures of *E*. *coli* AK in various catalytic states have shown that this kinase undergoes large conformational changes. At the two extremes, apo AK adopts an open conformation (PDB: 4ake) [8] whereas the inhibitor bound structure adopts a closed conformation (PDB: 1ake) [9] (Fig 2A) that differ by the two ligand binding domains/lids leading to an RMSD of 7.2 Å or TM score of 0.68 between the two crystal structures.

Input of the *E*. *coli* sequence for AK yields a relatively closed conformation for the fifteen AF2 models (Fig 2B) with the most disorder localized to the ATP binding lid. Scanning the structure identifies 21 sets of interacting residues yielding 315 additional AF2 models after MSA mutagenesis with three repeats per set of residues. Because targeting interaction surfaces with alanine substitutions could lead AF2 to generate models that are misfolded, we assessed the quality of the AF2 models for this test target by the TM score relative to the two crystal structures (Fig 2C). The broad range of TM scores spanning the range between the two structures is a remarkable demonstration that this method unlocks AF2’s ability to predict alternate conformations. However, we observed that some models do not appear to be a combination of the open and closed state as they fall below the dashed line, the TM score for the two structures relative to each other (Fig 2C). Close examination of these models reveals a variety of structural distortions that, in some cases, involved beta sheets being replaced by alpha helices (Fig A in S1 Text).

To explore whether an objective criterion can be applied to filter out such misfolded structures, we calculated the molprobity [10] score of all 330 models. The histogram for the molprobity score indicates that the majority of the scores lie between 1.6 and 1.9 with some tailing toward higher scores. (Fig 2D). To filter out models with high scores, which represent distorted structures, we calculated the average molprobity scores for the 22 sets of structures and then discarded sets where the average score was greater than one standard deviation of the mean relative to the median of all scores (Fig 2D). The resultant plot of TM scores for the remaining models indicate that the majority of models that were below the dashed line correspond to the sets with higher molprobity scores (Fig 2E). This suggests that this method can be used to systematically filter out sets with misfolded models.

While the relative TM scores highlight the conformational flexibility, this comparison requires the availability of more than one experimental structure. Alternatively, principal component analysis (PCA) provides a description of structural variance without multiple known protein structures. PCA for the AK crystal structures and the remaining AF2 models yields an interesting plot of principal components 1 and 2 (PC1, PC2) with one grouping of structures highly divergent from the rest of the models and the two crystal structures (Fig 2F). Examination of these structures indicate that while these structures passed the molprobity criteria, they are differently folded in the ATP binding lid compared to the crystal structures (Fig 2G). After removal of these misfolded models, PCA analysis of the remaining models yields two separate clusters suggestive of more than one intermediate between the open and closed structures (Fig 2H, Fig B in S1 Text). This set of models is consistent with a wide variety of studies on this canonical example of protein flexibility that indicate that the ATP binding lid and AMP binding lid are capable of opening and closing independently [11,12].

#### Ribose binding protein

**Fig 3:**
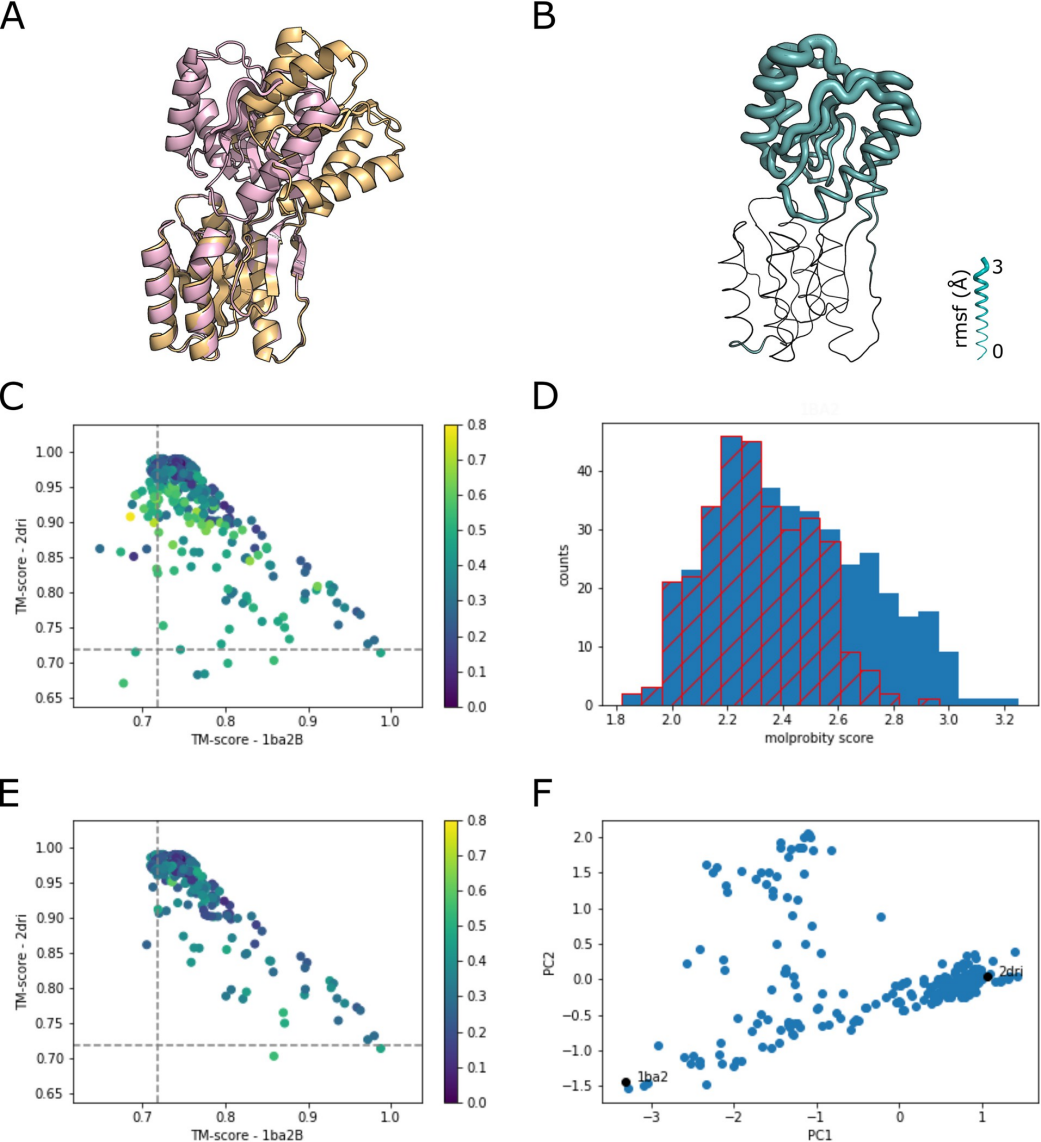
Sampling conformational flexibility of Ribose Binding Protein (RBP). A) Crystal structures of RBP: open, 1ba2B (lightorange) and closed, 2dri (lightpink). B) RBP model with the thickness of the chain based on the rmsf for the 15 AF2 models from the unmodified MSA. C) TM score plot comparing all AF2 models to the two crystal structures. The dashed line is the TM score between the two experimental structures. The color scale is based on the relative molprobity score, (MP–MPmin) / MPmin. D) Histogram of molprobity scores. In blue are all of the models. The hatched red plot is for the models parsed by excluding sets that are one standard deviation from the median of all models. E) TM score plot of the parsed set of models. F) PCA plot of the first two components for the parsed set of models.

Ribose binding protein (RBP) is a bacterial periplasmic protein involved in the chemotactic response to ribose. It has also been crystallized in a number of conformations including the ones compared here: the closed structure (PDB 2dri) [13] and an open structure (1ba2B) that was obtained as a consequence of the mutation D67R [14] (Fig 3A).

Similar to AK, input of the amino acid sequence for the Enterobacteriase RBP yields conformations that are most similar to the closed structure, 2dri (Fig 3B). Comparison of interacting residues within the sliding 11 amino acid window lead to 27 sets for mutational analysis of the MSA. The TM scores of the 420 AF2 models were calculated for comparison to the open and closed state of the two crystal structures (Fig 3C). Most of the models are near the closed state though there are several that yield a very high TM score with the open conformation. The molprobity score was calculated for all models and the sets that are one standard deviation of the mean greater than the median of all of the models were discarded (Fig 3D). The TM score plot supports this initial criteria for filtering as most of the models that lie outside of the progression of the closed to open states are eliminated (Fig 3E).

PCA of the remaining 315 AF2 models and the two crystal structures yields an apparent scatter plot of conformations with a cluster near the closed state (Fig 3F). The changes in the models lying between the two crystal structures is consistent with other studies of RBP [15,16]. The models within the scattered region with higher PC2 values are spread across several sets of mutations and do not appear to be misfolded (Fig C in S1 Text). Further experiments would be needed to ascertain whether these models have a role in RBP function.

The analysis of these two canonical examples of conformational flexibility indicate that the molprobity scores can be used as a first screen for misfolded models in the absence of any prior structures for the protein being examined. PCA can then be used as an additional screen for sets of models that do not fit the overall structural heterogeneity. In addition, the PCA allows for the identification and analysis of structures that might not be present in the structural database, leading to formulation of new hypotheses to be tested experimentally.

### Conformational diversity of membrane proteins with no structures in the Alphafold2 training set

We selected a set of membrane protein targets on the basis of a previous report examining the ability of AF2 to predict multiple conformations [7]. Experimental structures of these targets were determined subsequent to the training of AF2. The set includes members of diverse protein families (Table A in S1 Text) such as the major facilitator superfamily (MFS), LeuT-fold, cation diffusion facilitator (CDF), and G-protein coupled receptor (GPCR) family. The specific proteins are: MCT1 an MFS protein that requires an ancillary protein, basigin, for expression and transport of monocarboxylates; STP10 an MFS proton-sugar transporter from Arabidopsis thaliana; Lat1 a human amino acid transporter belonging to the LeuT-fold family; ZnT8 a dimeric, zinc transporter that is a member of the CDF; ASCT2 a sodium-dependent exchanger of neutral amino acids that forms a trimer and is thought to work via an elevator-type mechanism; CGRPR the GPCR for the calcitonin-gene-related peptide that is functionally part of a heterotrimer; PTH1R the GPRC for parathyroid hormone; and FZD7 the GPCR of the ’frizzled’ gene family that are receptors for Wnt signaling proteins. The modeling pipeline described above consisting of initial AF2 modeling, mutagenesis of the MSA, generation of new models from the mutated MSAs, molprobity structural filtering, and PCA analysis was applied to these membrane proteins. The modeling excluded accessary or interacting proteins. In addition, only the transmembrane spanning region is modeled for the GPCRs.

**Fig 4:**
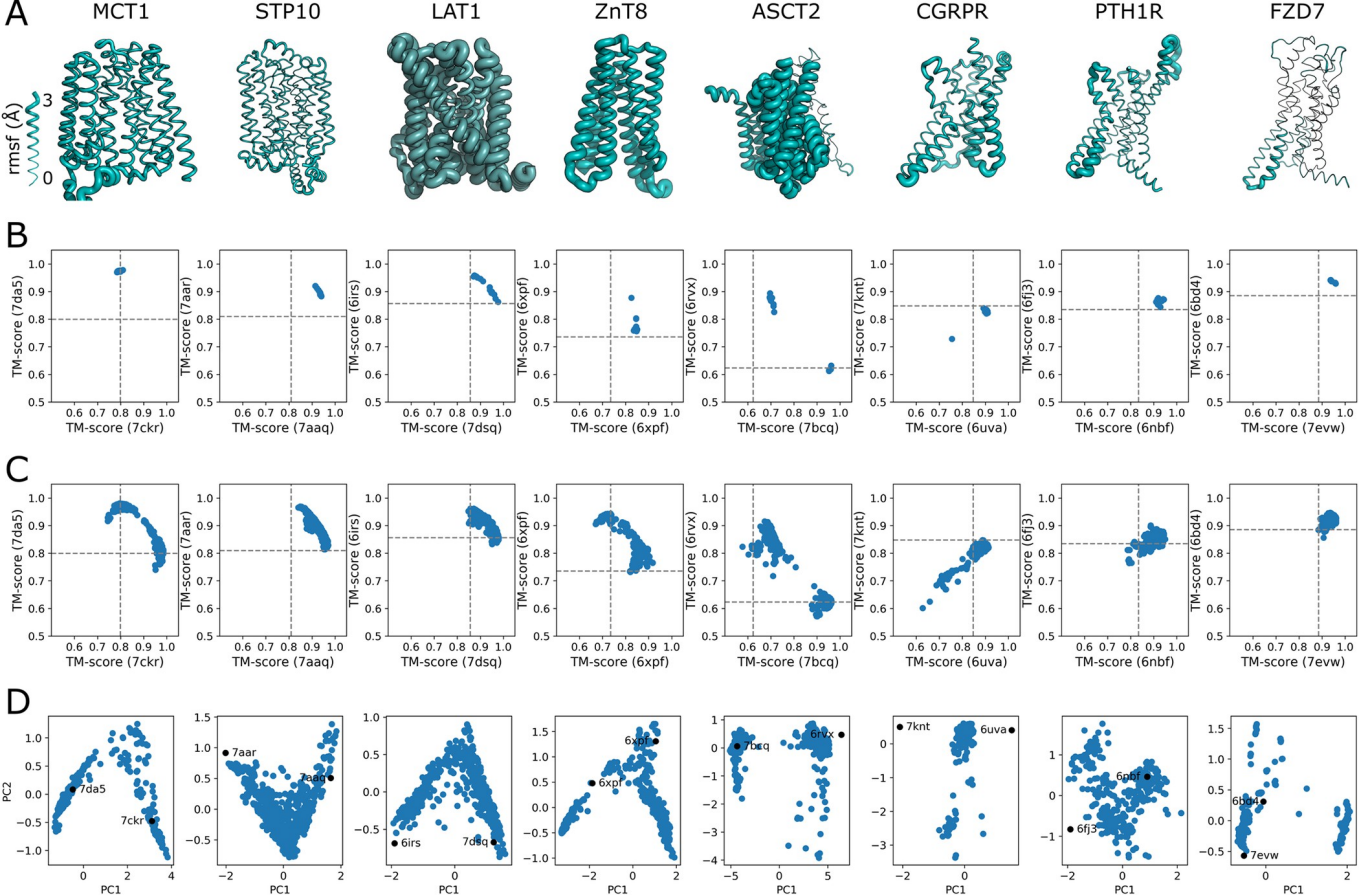
Sampling conformational flexibility of proteins with no structures in AF2 training set. A) Models with the thickness of the chain based on the rmsf for the 15 AF2 models from the unmodified MSA. B) TM score plot for the 15 initial models. C) TM score plot for all models after parsing for molprobity score. D) PCA of the parsed models. In Fig D in S1 Text are plots of the experimental structures and the best model based on TM score.

The modeling of the 8 membrane proteins using the unmodified MSA leads to a diverse breadth of conformational heterogeneity and correspondence with the now available experimental protein structures (Fig 4A–4B). The majority of the proteins adopt a single conformation with varying disorder within each conformation. Three of the proteins are able to adopt multiple conformations: ZnT8 exhibits one slightly different additional conformation; ASCT2 appears to adopt conformations near the two extrema exemplified by the experimental structures; Lat1 on the other hand appears to adopt conformations along the whole pathway between the two experimental structures. These results indicate that in some cases AF2 is directly able to yield multiple conformations without any modification of the MSA. This result is a consequence of two factors: the five different AF2 modelers are not monolithic and the increased variation of the MSA input as a result of varying the random seed. However, as seen below, the advantage of our systematic approach is that it is less dependent on the idiosyncrasies of the MSA, random seed, and the set of templates that were included in the training of AF2.

As performed for the canonical targets, mutational scanning of the MSA was carried out. These modified MSAs were then used by AF2 to generate new models. Following a molprobity filtering step, models were compared to the two experimental models (Fig 4C). For the five transporters, MCT1, STP10, Lat1, ZnT8, and ASCT2, the breadth of variability and correspondence to the two experimental structures increased in all cases (Fig E in S1 Text). We observed for the GPCRs, PTH1R and FZD7 but not CGRPR, an increase in the breadth of conformations that lie between the two experimental structures. In addition, the plots of the TM scores for the GPCRs were more diverse than the transporters. In the previous report using these targets, the authors were able to increase the number of apo-like structures for all of the GPCRs, but were unable to obtain alternate conformations of MCT1 without using a structural template [7].

To contextualize the multiple conformations without reference to the experimental structures, PCA was carried out on the parsed model sets. In some cases, additional sets or individual models were removed from the initial PCA. The plots of PC1 vs PC2 for the resulting analyses are shown in Fig 4D. Four of the transporters exhibit a V-shaped plot, supporting isomerization of the transporter between inward and outward facing conformations. Positions of the experimental structures relative to the AF2 models would suggest that not all of the experimental structures are at the extremes of the range of conformational heterogeneity. The plot for ASCT2 yields a gap between the two experimental conformations similar to the TM scores plot suggesting a lack of intermediate conformations. CGRPR exhibits only an intermediate state between the two experimental conformations in agreement with the TM scores plot, while PTH1R and FZD7 exhibit more diverse shapes relative to the transporters and each other. Overall, the PCA reflects the TM score plots, supporting the utility of the PCA in the absence of multiple experimental structures.

### Membrane proteins with one structure in the Alphafold2 training set

The previous report by Del Alamo et al. noted that their approach of using a shallow MSA was unable to generate more than one conformation for targets where one structure existed in the AF2 training set suggesting an intrinsic bias within AF2 [7]. Having established the applicability of our method to targets with no conformations in the training set, we examined whether this bias would be present here. Therefore, we selected the same set of targets for analysis by our method: the lipid II flippase MurJ and the multidrug and toxic compound extrusion (MATE) transporter PfMATE of the multidrug/oligosaccharidyl-lipid/polysaccharide (MOP) flippase superfamily; SERT the human serotonin transport belonging to the LeuT-fold family; and CCR5 a GPCR for CC chemokines.

**Fig 5:**
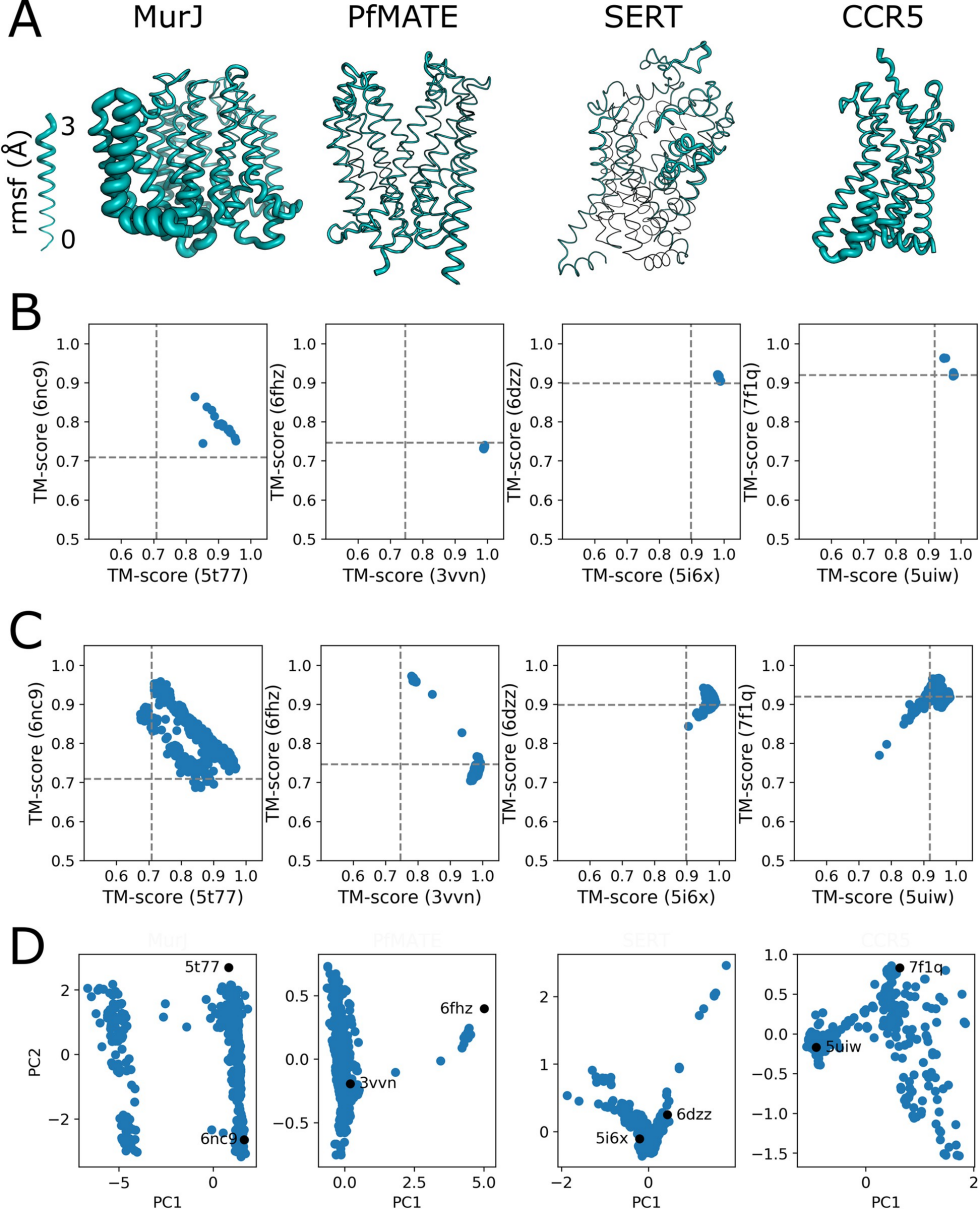
Sampling conformational flexibility of proteins with one structure in AF2 training set. A) Models with the thickness of the chain based on the rmsf for the 15 AF2 models from the unmodified MSA. B) TM score plot for the 15 initial models. C) TM score plot for all models after parsing for molprobity score. D) PCA of the parsed models. In Fig F in S1 Text are plots of the experimental structures and the best model based on TM score.

Following the pipeline established above, the sequences of these four membrane proteins were used to predict models by AF2 (Fig 5A–5B). The initial models for MurJ are fairly diverse with a wide variance for TM13-14, whereas PfMATE, another member of the MOP superfamily, has essentially one conformation, outward-open that is similar to that present in the training set. It should be noted that while the full length protein was modeled, the analysis for PfMATE does not include TM1 as it is unraveled in the low pH experimental structure (determined subsequent to the training of AF2) as a result of crystal contacts and does not appear to represent the native conformation [17]. The serotonin transporter, SERT, has some variability in TMs 1b and 6a, which are a part of the opening and closing of the extracellular vestibule. CCR5 exhibits more variability than the GPCRs that had no structure in the training set.

Mutational scanning of the MSA and AF2 model generation were carried out for these four proteins. After filtering by molprobity scores, the resulting AF2 models were compared to the experimental structures (Fig 5C). MurJ has an interesting profile and the bifurcation of the TM scores is related to the highly diverse transmembrane helices 13–14 since analysis of the TM scores without this region yields a single line of models between the two structures (Fig G in S1 Text). This is particularly notable as transmembrane helices 13–14 are thought to be the site of binding for the lipid II isoprenoid tail [18]. Conversely, there are hardly any additional conformations for PfMATE, but there are several models that do represent the alternate inward-open conformation that was not present in the AF2 training template set. SERT does adopt alternate conformations, though the structural changes are not completely in the direction of the second experimental conformation. Similar to the GPCRs with no structures in the training set above, mutational analysis for CCR5 generates a more diverse set of models. The generation of alternate conformations for these four proteins argues against a general bias as the origin of the observation that decreasing the number of sequences in the MSA did not lead to the alternate conformations for these proteins.

PCA of this set of proteins leads to a diverse set of plots (Fig 5D). The MurJ plot of the first two principal components yields a square shape, comparable with the bifurcated TM score plot; while the data without transmembrane helices 13–14 exhibit a V-shape plot for the principal components (Fig G in S1 Text). If the AF2 models generated here for the transmembrane helices 13–14 region are correct, this would suggest that there is coupling between ion gradients and movement of transmembrane helices 13–14 and by proxy the movement of lipid across the membrane. The small number of alternate conformations for PfMATE is reflected in the plot of PC1 vs PC2. Interestingly, the plot would suggest that the experimental structure, 3vvn, is not the fully outward-open state. If the AF2 models for SERT are correct, then there are more conformations at the extrema for the transport cycle of serotonin. For CCR5 there is a line of models from the two experimental conformation with a scattering away from them. This scatter is reflected in the TM score plots. These results further support the utility of the PCA as a tool for classifying models in the absence of multiple experimental structures.

### Conformational flexibility by targeted in silico mutagenesis

Whereas the protocol above makes no assumption regarding the surfaces to be mutated, prior knowledge of the underlying model of conformational changes may restrict the search to the hypothesized areas of contacts. We have carried this out for the MOP family transporter PfMATE and the multidrug transporter LmrP.

Based on the two-fold symmetry for PfMATE we hypothesized that the interface between the two halves would mediate the change in conformation. Therefore, the residues that were within 4 Å between the N- and C-terminal halves of the protein were mutated to alanine in the MSA as before and new AF2 models were generated. These residues lead to a complete reversal of conformation from the outward-open for all of the initial models to inward-open for all of the models generated from the mutated MSA (Fig H in S1 Text).

Directed mutation of the MSA was carried out for LmrP, a target in CASP XIV. Modeling of the LmrP structure with the unmodified MSA yields mostly inward-open conformations (Fig I1 in S1 Text), a result that differs from the initial models obtained by Deepmind that were all inward-open conformations. The difference is most likely due to the update (v 2.1) of the model parameters for Alphafold used here. To obtain an alternate outward facing conformation, the Deepmind team parsed the MSA and limited the structural templates to those that are outward facing [6]. In contrast, the approach here does not use structural templates. Rather, the residues that mediate the interface between the two halves of the protein in the inward facing AF2 model were explored. The mutations targeted residues in both the N- and C-terminal half, residues in either the N- or C-terminal half, and a set of 3 residues at the center of the transmembrane region. These mutations prompted AF2 to generate mostly outward-open conformations that match the experimental structure (Fig I1 in S1 Text). PCA was carried out on the AF2 models and the single experimental structure and yielded a mostly V-shaped curve as seen for the other transporters (Fig I2 in S1 Text). The four structures that were not inward-open in the initial runs of AF2 do not segregate with any of the other models and appear to be partially open on both sides (Fig I3 in S1 Text) suggesting that these are misfolded structures. The ability to generate the opposite conformations with fewer modified MSAs supports the use of directly targeting residues to mutate in the MSA based on prior knowledge of conformational changes.

## Discussion

The premise of the work presented here is that the MSA contains information on multiple protein conformations. Therefore, it follows that AF2 can generate these conformations with appropriate modifications of the MSA. Our method entails simple *in silico* mutagenesis to successfully coerce AF2 to sample alternative conformations for a number of target proteins both water-soluble and membrane embedded (Figs 2–5). This simple *in silico* mutagenesis is followed by two filtering steps to remove misfolded proteins. The molprobity score sets a threshold to filter out sets of models that overall yield poorer quality structures while the PCA enables the removal of models that are not part of an ensemble. In addition, the PCA allows for the development of specific hypothesis regarding the conformational dynamics in the absence of experimental structures.

Our mutagenesis method requires no prior structural knowledge about the protein of interest or its conformational landscape. The protein sequence is used to generate five models by AF2. Typically, the top ranked model is probed to identify contact points within the structure, though any of the five models can be used. Contact sites between the rest of the protein and residues within a chosen window are determined. These contact residues are then mutated to alanine in the MSA and new models are generated. The window of interest is scanned along the protein sequence leading to the generation of sets of models. If the alanine substitutions do not alter the information content of the MSA, AF2 returns similar models to the wild-type sequence. In contrast, if the mutations to alanines do alter the information content of the MSA, the AF2 models are expected to include alternate conformations. Our underlying hypothesis is that these models reflect the protein conformation space because the MSA encodes this information. This is supported by a study that found that the distance contact map generated from co-evolution analysis is correlated to the flexibility of the residue pairs [19]. That alternate conformations are encoded in the MSA and the ability of the method to yield multiple secondary structural elements (Fig A in S1 Text) would support this methodology in examining fold-switch proteins where the standard AF2 pipeline was generally unable to model both conformations [20].

One variable in this method is the size of the window for alanine scanning. The choice of 11 amino acid presented here was selected based of the expected size of the structural elements to be probed, mainly long α-helices of transmembrane proteins, and the number of models that would be generated, i.e. the protein length divided by the window length. As the length of the window gets smaller, more models need to be generated and the compute time of the AF2 calculations increases. To explore the effect of window size on the ensemble of models, we tested the effect of smaller windows of 8 and 5 amino acid on adenylate kinase. The PCA of the resulting models from the three window sizes show good overlap of the conformational space explored (Fig J in S1 Text). These results support the use of the 11 amino acid window, though a smaller window might be more appropriate when the structural elements of the protein of interest are smaller or similar in size to the length of this window.

A question with this method is the effect of mutating residues to alanine on a protein that does not undergo a conformational change. We tested this question by using a 3 amino acid window on the protein ubiquitin. The small window size was selected because ubiquitin is at least 1/4 as large compared to the membrane proteins that make up the testing set. The same protocol was followed where the MolProbity score is used to filter out the initial set of models (Fig K1 in S1 Text). The remaining models were further examined by PCA along with several sets of structures obtained by NMR spectroscopy (Fig K2 in S1 Text). We observed a slight shift between the center of the AF2 models and the center of the NMR structures, which is most likely due to subtle differences in the AF2 model compared to the experimental structure (Fig K3 in S1 Text). However, the strong overlap in the PCA indicates that structural flexibility is highly similar (Fig K4 in S1 Text) and examination of the structures on the periphery do not indicate any additional conformations. The similarity in the explored conformational space and lack of any new conformations generated by AF2 further supports the overall conclusion that the method presented here, when filtered for misfolded models, does not lead to gross deviations from experimentally sampled conformations.

When the alternate methodology of subsampling the MSA was released in preprint [21], an additional, complementary method was suggested in a Twitter feed [22]. This method entailed using dropout and altering the random seed within AF2 to sample a protein’s conformational space. It appears to work for one case, but was unsuccessful for MCT1. While it was mentioned that combining dropout and subsampling of the MSA might be of interest [22] nothing further has been put forth. Here we have carried out a comparison with the subsampling method by examining all of the proteins used in the now published study [7]. Plots of the TM-score for the AF2 models generated by making *in silico* mutations in the MSA suggest that the approach described here is capable of generating both conformations for all of the targets, except CGRPR, including those where the MSA subsampling method failed (Fig 4–5). This demonstrates the general utility of our methodology in generating ensembles of multiple conformations regardless of whether the protein is in the training set.

Alternate conformations for almost all of the proteins were obtained and in some cases without modifying the MSA. These results support that the five different AF2 modelers are not monolithic and the first step in obtaining alternate conformations are multiple runs of AF2 with different random seeds. The inconsistent ability of AF2 to generate multiple conformations with the unmodified MSA and the failures of both methodologies might have similar roots, the information content of the MSA. This would explain the disparate results and how different random seeds lead to multiple conformations. This is supported by a recent report examining optimizing MSAs for maximizing AF2 model confidence, though the changes that lead to higher confidence were self-inconsistent [23]. How the makeup of the MSA, such as generated via MMseq2 or through the AF2 pipeline, affects the alternate conformations obtained by AF2 requires further examination. Regardless of the source of the MSA, the methodology developed here is consistent with the notion that AF2 did not just learn structures, but is able to generate multiple conformations rooted in physical principles. This is supported by results that showed that AF2’s pLDDT score is highly correlated to model accuracy [24], though the learned physical principles do not describe protein folding pathways [25].

The results presented here strongly support manipulation of the MSA to generate ensembles of multiple conformations of proteins via AF2. Furthermore, the PCA of the ensembles and conformations presented fully support the biochemical significance of the models generated here. These *in silico* structures can guide experimental design and be tested using spectroscopic approaches, and will ultimately provide a framework for interpretation of existing biochemical data and the development of mechanistic models.

## Methods

### Initial Alphafold2 structure

Protein sequences were downloaded as fasta files from NCBI [26]. These sequences were used as input for colabfold_batch that is part of ColabFold [27]. ColabFold implements folding of the protein with the models for Alphafold2 using MMseqs2 to generate the MSA. The models were generated with the default parameters, which includes no template [3,27–30]. The rationale for not including any templates is to allow Alphafold2 to generate structural intermediates that may not be achieved by the bias of including structural templates.

### In silico mutagensis

The total length of the protein to be alanine-scanned was determined from the pLDDT results for the top AF2 model, though if the initial AF2 models have different conformations any of the models could be chosen. Both the N- and C- terminal ends were truncated where the pLDDT values were less than the mean of all the pLDDT values. An 11 amino acid window was scanned from these starting and ending points. Within this window, all interacting residue pairs of this region and the rest of the protein that lie within 4 Å of each other are tabulated. To keep from destabilizing secondary structure, any of the interacting partners that were within 4 amino acids in the primary sequence relative to the region of interest were omitted. Four angstroms was chosen as the cutoff to encompass polar and ionic interactions including those mediated by water. Alternate cutoffs or criteria could be chosen to yield smaller or larger sets of potentially interacting pairs.

### Modification of the MSA

The initial MSA generated by MMseqs2 is in a3m format. This format has the query sequence in all capitals without gaps. The subsequent sequences have a dash for a gap in that sequence and lowercase letters for a gap in the first sequence. The alanine substitutions were made in the equivalent amino acid position across all sequences. This substitution was not made if the equivalent position was a gap. The choice of alanine is to minimize any negative consequences of the mutation on secondary structure. A jupyter notebook to carry out this mutagenesis step is available (https://github.com/RSvan/SPEACH_AF).

### Additional Alphafold2 runs

The subsequent runs using the modified sequence and MSA were again carried out with colabfold_batch, 3 times for each MSA for a total of 15 models for each 11 aa window examined. A modification was made to batch.py within ColabFold to allow for a different random seed for each run increasing the variability in the modelers of Alphafold2. The modified batch.py file is available (https://github.com/RSvan/SPEACH_AF).

### Filtering of Alphafold2 models

The AF2 models were subjected to molprobity scoring [10]. The median and the standard deviation of the mean (SDM) for all models were calculated. Based on the mutagenesis sliding window the mean molprobity score was calculated for each set of 15 models. The sets where the mean was greater than one SDM from the median were parsed out from the total set. The parsed set of models underwent principal component analysis (PCA) with ProDy [31]. The plots for principal component 1 (PC1) vs principal component 2 (PC2) were plotted. This plot was then used for manual inspection for outliers relative to the rest of the data. These outliers were removed from the set and the PCA was repeated until there were no apparent outliers.

### Additional analysis

The relative molprobity score (MP) was calculated by (MP-MP_min_)/MP_min_. The TM score was obtained with TM_align [32]. The root mean square fluctuation (rmsf) was calculated with ProDy [31]. Figure generation was carried out in Pymol [33].

## Supporting information

S1 Text Contains all supplemental Tables and Figures. Table A: Membrane Protein Structures. Fig A: Examples of Misfolded Adenylate Kinase. Fig B: Structural intermediates of Ribose Binding Protein. Fig C: Structures outside the transition from 2dri to 1ba2 are not misfolded. Fig D: Plots matching models to experimental structures. Fig E: Conformation diversity of the models. Fig F: Plots matching models to experimental structures. Fig G: Analysis of MurJ without transmembrane helices 13 and 14. Fig H: PfMATE conformational flexibility. Fig I: LmrP conformational flexibility. Fig J: PCA of Adenylate Kinase. Fig K: SPEACH_AF on ubiquitin.
(PDF)
