# Supplementary material for: SPEACH_AF: Sampling protein ensembles and conformational heterogeneity with Alphafold2
Source: PLoS Comput Biol. 2022 Aug 22;18(8):e1010483. doi: 10.1371/journal.pcbi.1010483 (PMC9436118; doi:10.1371/journal.pcbi.1010483)
Supplement: S1 Text — Table A: Membrane Protein Structures. Fig A: Examples of Misfolded Adenylate Kinase. Fig B: Structural intermediates of Ribose Binding Protein. Fig C: Structures outside the transition from 2dri to 1ba2 are not misfolded. Fig D: Plots matching models to experimental structures. Fig E: Conformation diversity of the models. Fig F: Plots matching models to experimental structures. Fig G: Analysis of MurJ without transmembrane helices 13 and 14. Fig H: PfMATE conformational flexibility. Fig I: LmrP conformational flexibility. Fig J: PCA of Adenylate Kinase. Fig K: SPEACH_AF on ubiquitin. (PDF) [file pcbi.1010483.s001.pdf]

Supporting Information for

SPEACH_AF: Sampling protein ensembles and conformational heterogeneity with Alphafold2

Richard A. Stein and Hassane S. Mchaourab

Department of Molecular Physiology and Biophysics, Vanderbilt University

Table A: Membrane Protein Structures		
	Outward-facing/active	Inward-facing/inactive
MCT1	7ckr [1]	7da5 [1]
STP10	7aaq [2]	7aar [2]
Lat1	7dsq	6irs [3]
ZnT8	6xpf_A [4]	6xpf_B [4]
ASCT2	7bcq [5]	6rvx [6]
CGRPR	6uva [7]	7knt [8]
PTH1R	6nbf [9]	6fj3 [10]
FZD7	7evw [11]	6bd4 [12]
	In training set	Not in training set
MurJ	5t77 [13]	6nc9 [14]
PfMATE	3vvn [15]	6fhz [16]
SERT	5i6x [17]	6dzz [18]
CCR5	5uiw [19]	7f1q [20]

1. Wang N, Jiang X, Zhang S, Zhu A, Yuan Y, Xu H, et al. Structural basis of human monocarboxylate transporter 1 inhibition by anti-cancer drug candidates. Cell. 2021;184: 370-383.e13. doi:10.1016/j.cell.2020.11.043

2. Bavnhøj L, Paulsen PA, Flores-Canales JC, Schiøtt B, Pedersen BP. Molecular mechanism of sugar transport in plants unveiled by structures of glucose/H+ symporter STP10. Nat Plants. 2021;7: 1409–1419. doi:10.1038/s41477-021-00992-0

3. Yan R, Zhao X, Lei J, Zhou Q. Structure of the human LAT1–4F2hc heteromeric amino acid transporter complex. Nature. 2019;568: 127–130. doi:10.1038/s41586-019-1011-z

4. Xue J, Xie T, Zeng W, Jiang Y, Bai X. Cryo-EM structures of human ZnT8 in both outward- and inward-facing conformations. Boudker O, Penmatsa A, editors. eLife. 2020;9: e58823. doi:10.7554/eLife.58823

5. Garibsingh R-AA, Ndaru E, Garaeva AA, Shi Y, Zielewicz L, Zakrepine P, et al. Rational design of ASCT2 inhibitors using an integrated experimental-computational approach. PNAS. 2021;118. doi:10.1073/pnas.2104093118

6. Garaeva AA, Guskov A, Slotboom DJ, Paulino C. A one-gate elevator mechanism for the human neutral amino acid transporter ASCT2. Nat Commun. 2019;10: 3427. doi:10.1038/s41467-019-11363-x

7. Liang Y-L, Belousoff MJ, Fletcher MM, Zhang X, Khoshouei M, Deganutti G, et al. Structure and Dynamics of Adrenomedullin Receptors AM1 and AM2 Reveal Key Mechanisms in the Control of Receptor Phenotype by Receptor Activity-Modifying Proteins. ACS Pharmacol Transl Sci. 2020;3: 263–284. doi:10.1021/acsptsci.9b00080

8. Josephs TM, Belousoff MJ, Liang Y-L, Piper SJ, Cao J, Garama DJ, et al. Structure and dynamics of the CGRP receptor in apo and peptide-bound forms. Science. 2021;372: eabf7258. doi:10.1126/science.abf7258

9. Zhao L-H, Ma S, Sutkeviciute I, Shen D-D, Zhou XE, de Waal PW, et al. Structure and dynamics of the active human parathyroid hormone receptor-1. Science. 2019;364: 148–153. doi:10.1126/science.aav7942

10. Ehrenmann J, Schöppe J, Klenk C, Rappas M, Kummer L, Doré AS, et al. High-resolution crystal structure of parathyroid hormone 1 receptor in complex with a peptide agonist. Nat Struct Mol Biol. 2018;25: 1086–1092. doi:10.1038/s41594-018-0151-4

11. Xu L, Chen B, Schihada H, Wright SC, Turku A, Wu Y, et al. Cryo-EM structure of constitutively active human Frizzled 7 in complex with heterotrimeric Gs. Cell Res. 2021;31: 1311–1314. doi:10.1038/s41422-021-00525-6

12. Yang S, Wu Y, Xu T-H, de Waal PW, He Y, Pu M, et al. Crystal structure of the Frizzled 4 receptor in a ligand-free state. Nature. 2018;560: 666–670. doi:10.1038/s41586-018-0447-x

13. Kuk ACY, Mashalidis EH, Lee S-Y. Crystal structure of the MOP flippase MurJ in an inward-facing conformation. Nat Struct Mol Biol. 2017;24: 171–176. doi:10.1038/nsmb.3346

14. Kuk ACY, Hao A, Guan Z, Lee S-Y. Visualizing conformation transitions of the Lipid II flippase MurJ. Nat Commun. 2019;10: 1736. doi:10.1038/s41467-019-09658-0

15. Tanaka Y, Hipolito CJ, Maturana AD, Ito K, Kuroda T, Higuchi T, et al. Structural basis for the drug extrusion mechanism by a MATE multidrug transporter. Nature. 2013;496: 247–251. doi:10.1038/nature12014

16. Zakrzewska S, Mehdipour AR, Malviya VN, Nonaka T, Koepke J, Muenke C, et al. Inward-facing conformation of a multidrug resistance MATE family transporter. PNAS. 2019;116: 12275–12284. doi:10.1073/pnas.1904210116

17. Coleman JA, Green EM, Gouaux E. X-ray structures and mechanism of the human serotonin transporter. Nature. 2016;532: 334–339. doi:10.1038/nature17629

18. Coleman JA, Yang D, Zhao Z, Wen P-C, Yoshioka C, Tajkhorshid E, et al. Serotonin transporter–ibogaine complexes illuminate mechanisms of inhibition and transport. Nature. 2019;569: 141–145. doi:10.1038/s41586-019-1135-1

19. Zheng Y, Han GW, Abagyan R, Wu B, Stevens RC, Cherezov V, et al. Structure of CC Chemokine Receptor 5 with a Potent Chemokine Antagonist Reveals Mechanisms of Chemokine Recognition and Molecular Mimicry by HIV. Immunity. 2017;46: 1005-1017.e5. doi:10.1016/j.immuni.2017.05.002

20. Zhang H, Chen K, Tan Q, Shao Q, Han S, Zhang C, et al. Structural basis for chemokine recognition and receptor activation of chemokine receptor CCR5. Nat Commun. 2021;12: 4151. doi:10.1038/s41467-021-24438-5

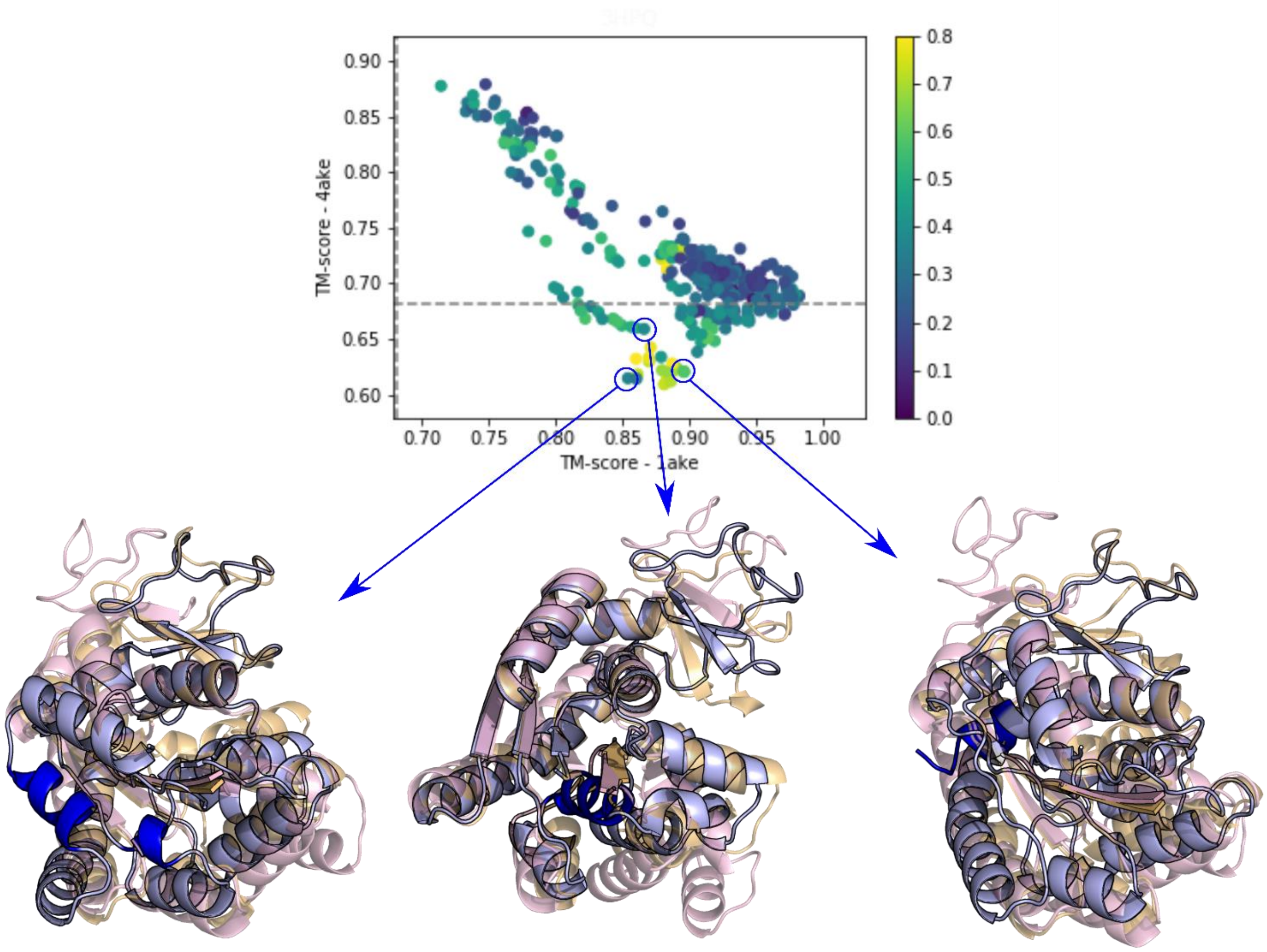

Fig A. Examples of Misfolded Adenylate Kinase. All three examples have alpha-helices (blue) in place of what are beta-sheets in the experimental structures. On the right the N-terminal residues are on the opposite side of the protein, but yet the overall fold fits very well to the experimental structure.

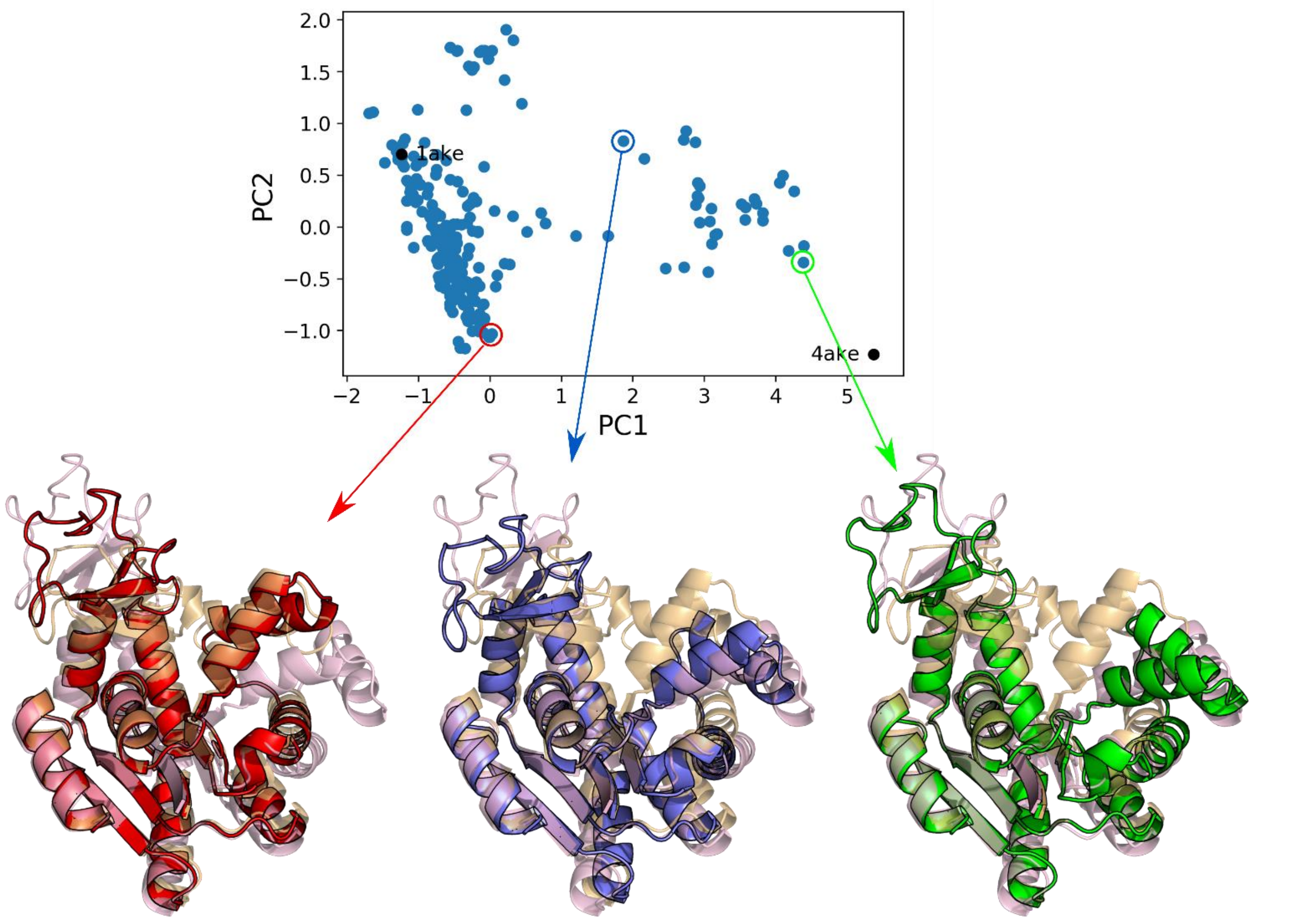

Fig B. Structural intermediates of Ribose Binding Protein. Examples highlighting the independent movement of the ATP and AMP lids. On the left and right are structures with the ATP lid more open than the 1ake structure. The middle and right structures have an open AMP lid.

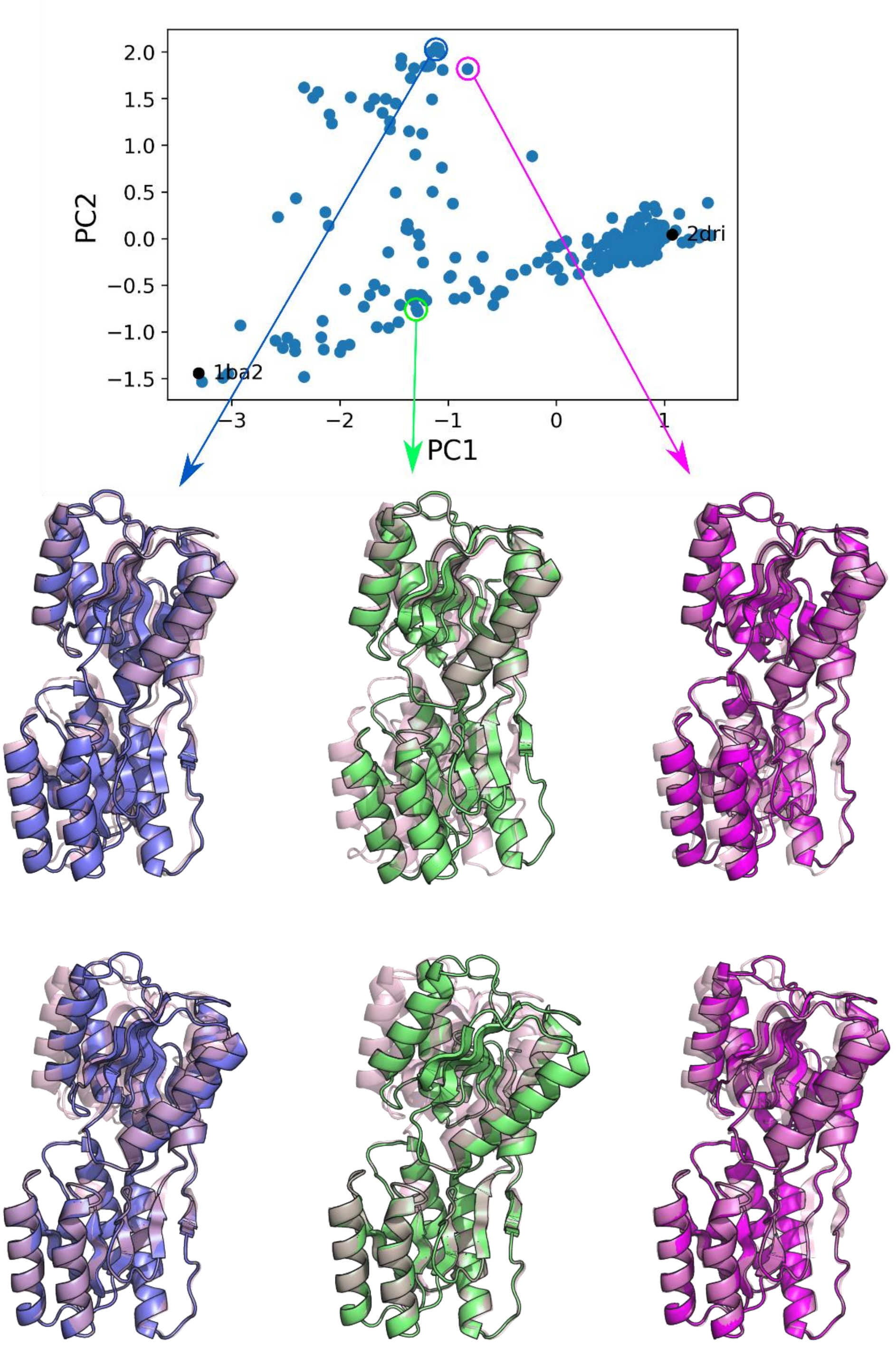

Fig C. Structures outside the transition from 2dri to 1ba2 are not misfolded. Upper structures are aligned based on the upper domain of 2dri. Lower structures are aligned based on the lower domain of 2dri. There is comparable alignment of the lower and upper domains compared to the model that is intermediate of 2dri and 1ba2 (green). The difference in the structures is the orientation of the two domains relative to each other.

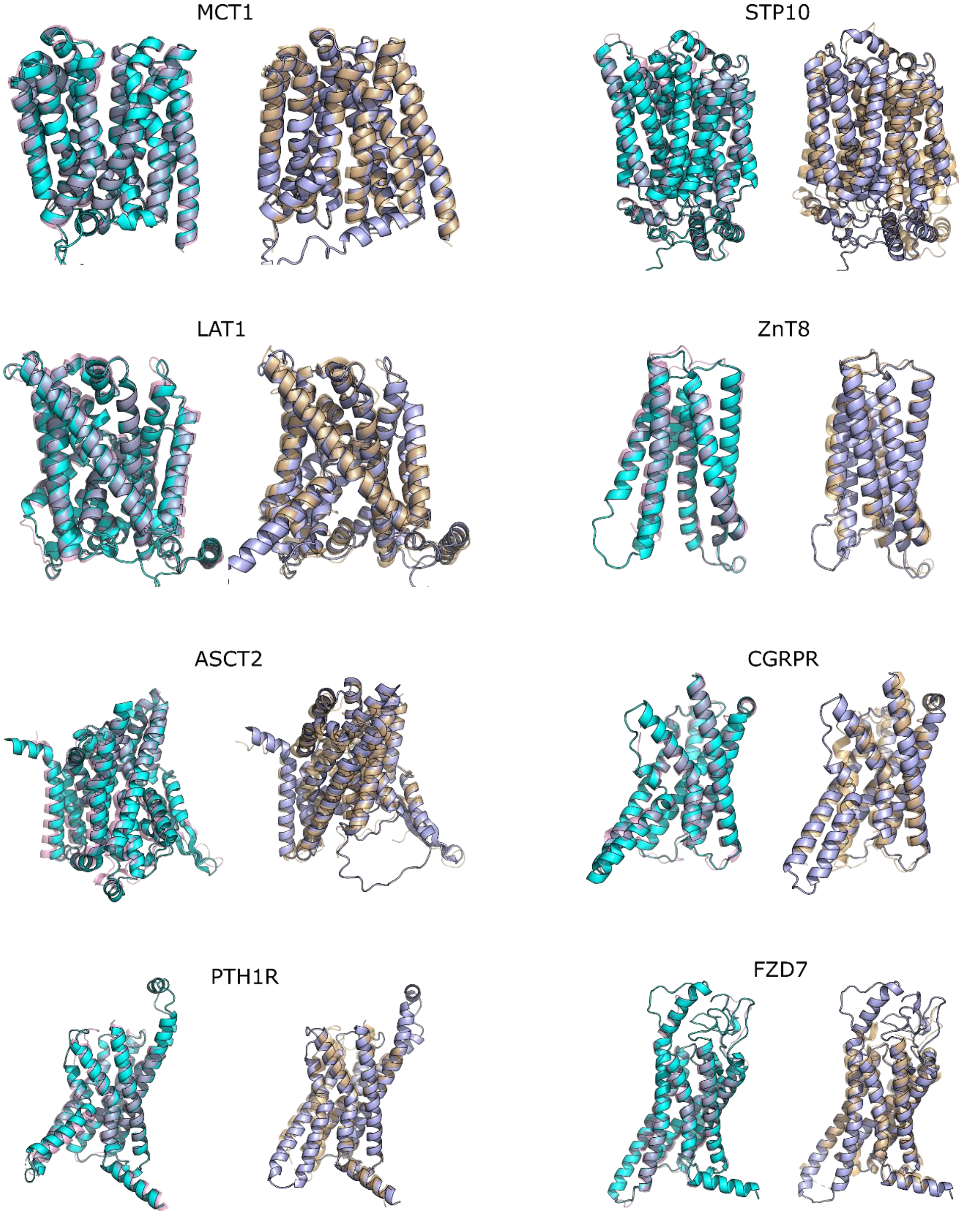

Fig D. Superposition of AF2 models to experimental structures. On the left is the model with the highest TM score overlayed with the outward-facing or active conformation. On the right is the model with the highest TM score to the inward-facing or inactive conformation.

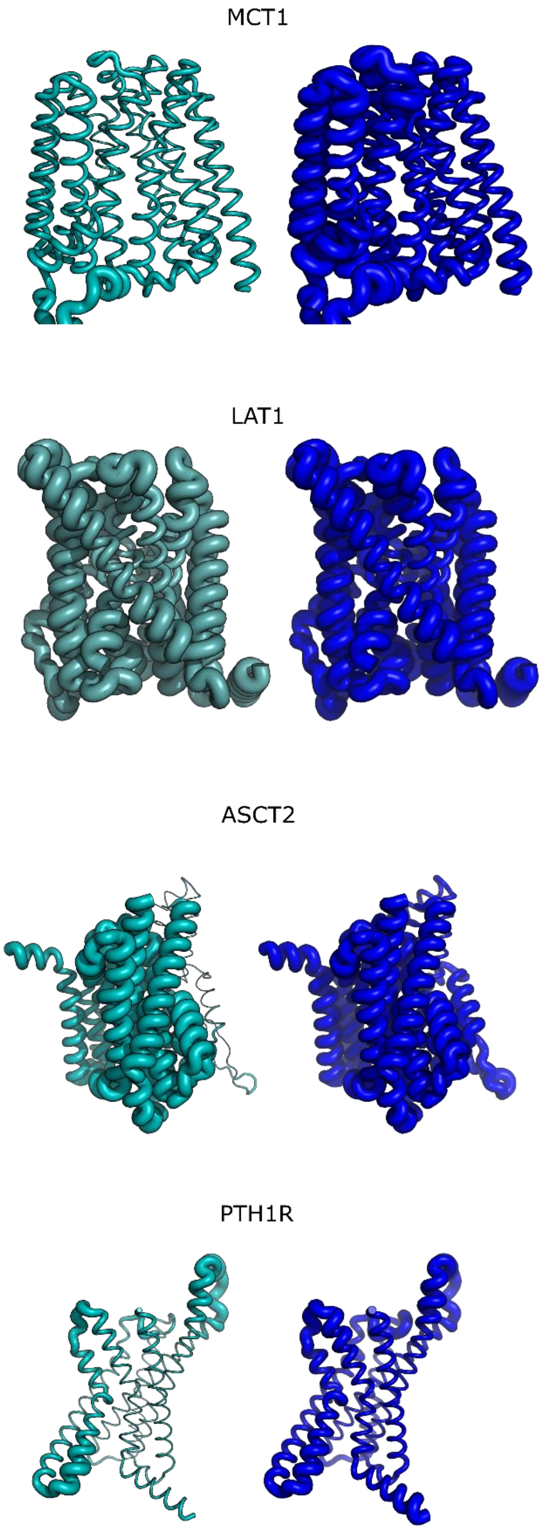

Fig E. Conformation diversity of AF2 models. On the left (teal) for each pair is model and disorder from the initial 15 AF2 models shown in Fig. 4. On the right (blue) is the same model with the disorder from the complete set of parsed models.

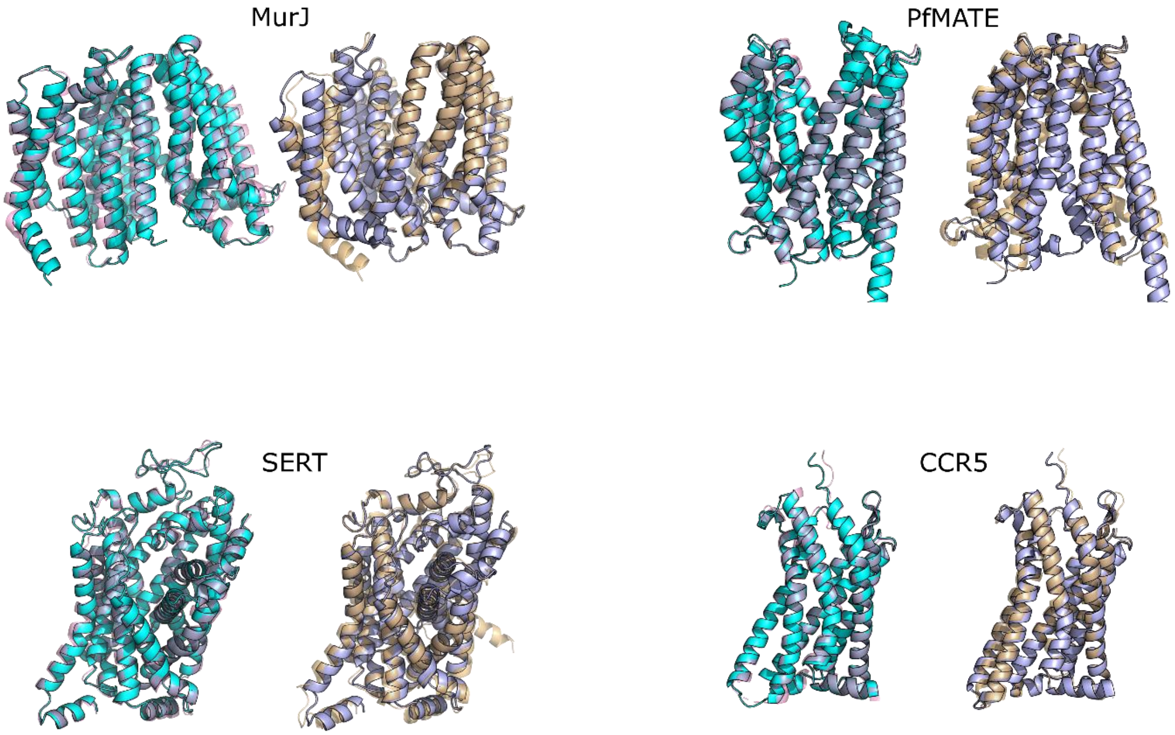

Fig F. Superposition of AF2 models to experimental structures. On the left is the model with the highest TM score overlayed with the outward-facing or active conformation. On the right is the model with the highest TM score to the inward-facing or inactive conformation.

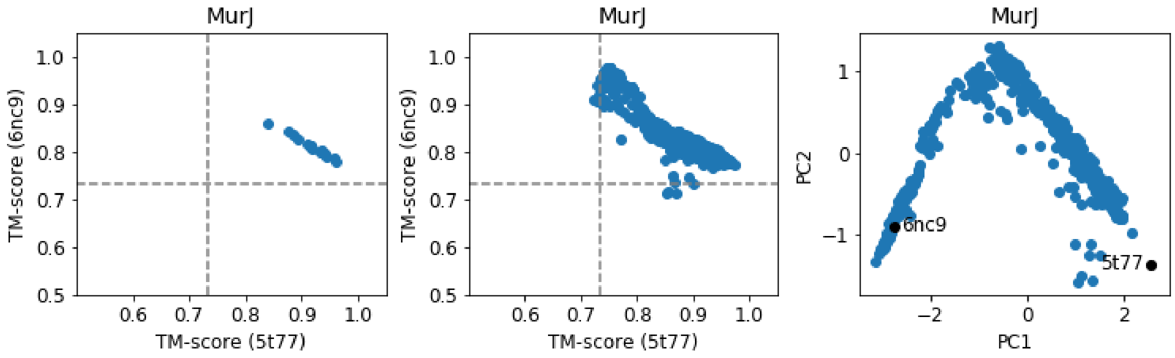

Fig G. Analysis of MurJ without transmembrane helices 13 and 14. Left) The TM score plots for the initial 15 models. Center) The TM score plot for the parsed models. Right) Plot of the first two principal components from the PCA. All of these plots lose the bifurcation that suggests a potential conformational role for transmembrane helices 13 and 14 in lipid transport.

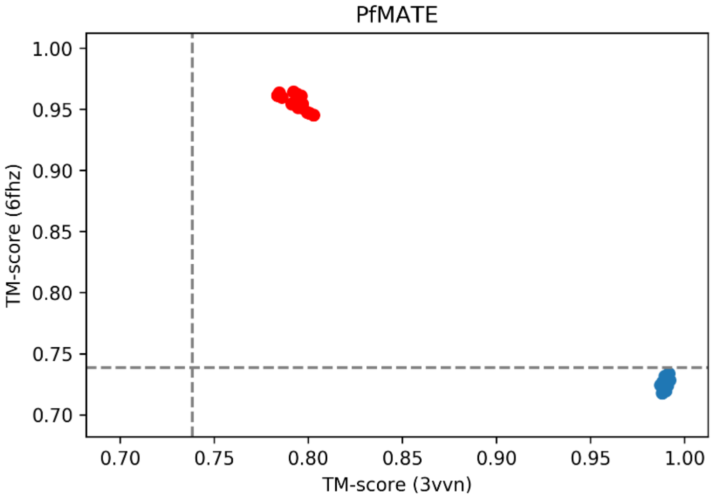

Fig H. PfMATE conformational flexibility. The TM score for the models from the initial (blue) and modified MSA (red). All of the models from the modified MSA are inward-open opposite from the initial models.

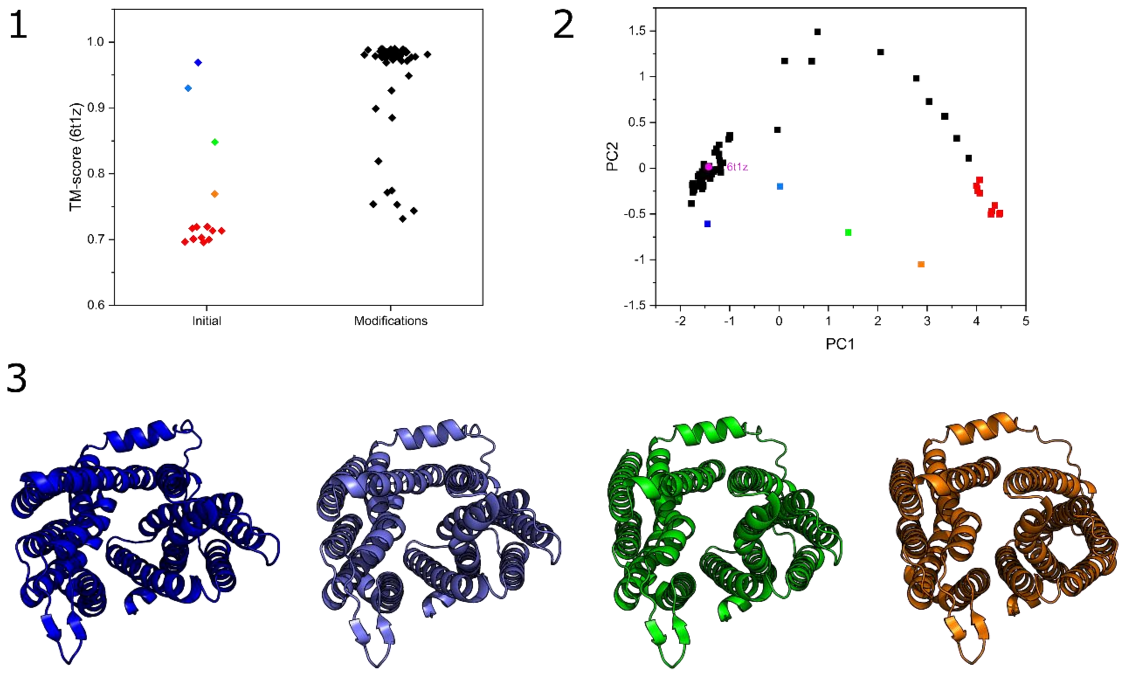

Fig I. LmrP conformational flexibility. 1) The TM-scores for the initial 15 models and for the rest of the models compared to the single experimental structure. 2) PCA analysis plot. The four models from the initial AF2 run that do not cluster with the rest of the models deviate from the rest of the ensemble of structures. 3) View from the extracellular vestibule highlighting the more open conformation for these models.

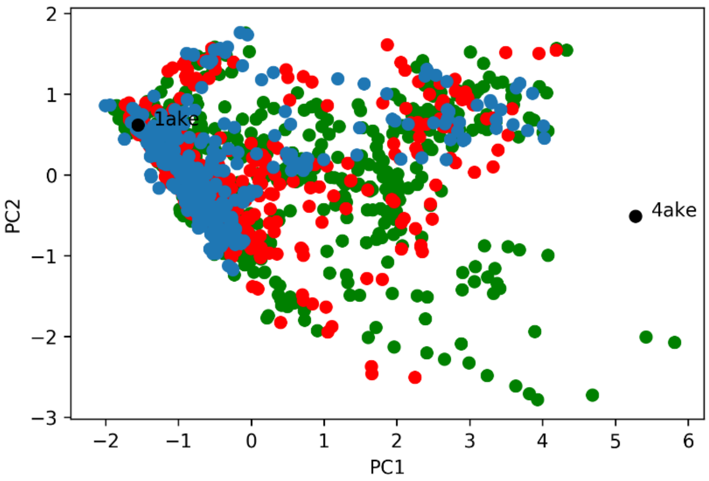

Fig J. PCA of Adenylate Kinase. Combined PCA of Adenylate kinase models using different size mutagenesis windows: 11 amino acids (blue), 8 amino acids (red), and 5 amino acids (green). The three window sizes yield similar information about the structural elements that are conformationally flexible. There is an increase in flexibility with smaller windows supporting using a smaller window when the conformational changes are in smaller structural elements.

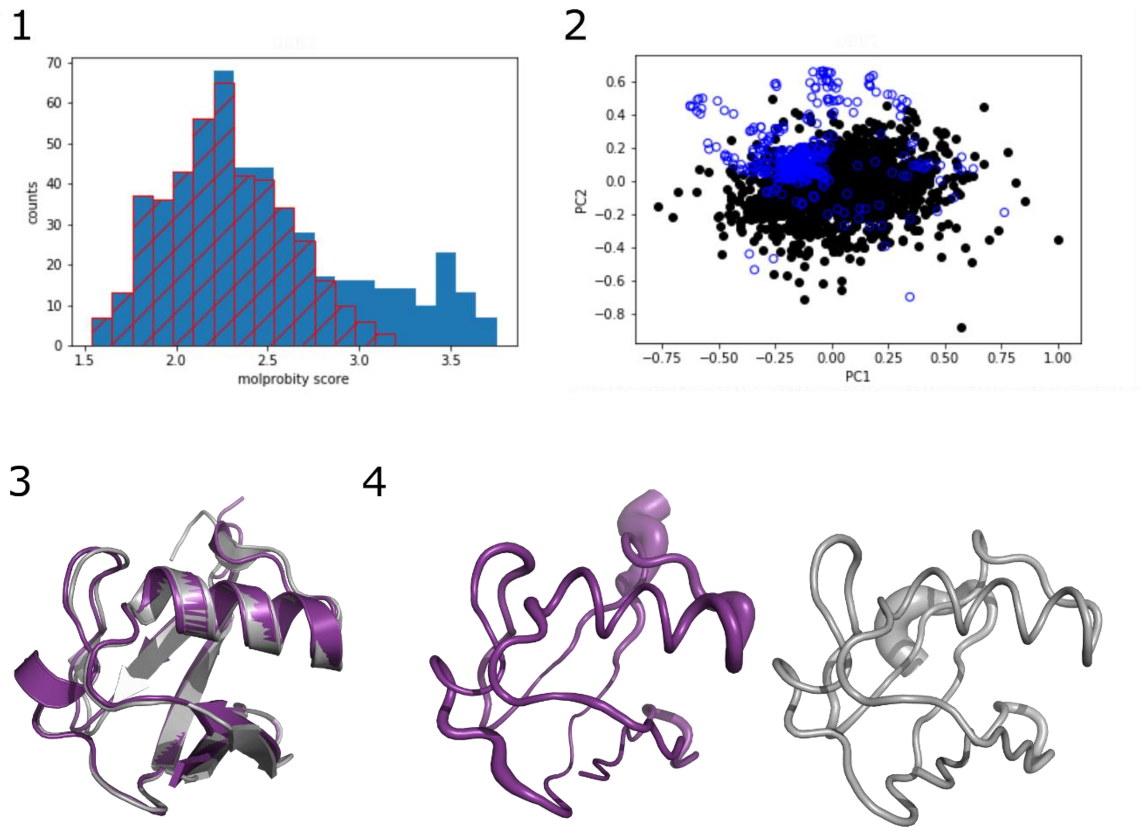

Fig K. SPEACH_AF on ubiquitin. 1) Histogram plot of the MolProbity scores for ubiquitin using a 3 amino acid window. 2) PCA analysis for the parsed AF2 models (blue circles) and NMR structures (black circles; PDBs: 1d3z, 1g6j, 1v80, 1v81, 1xqq, 2kn5, 2kox, 2k39, 2lj5, 2nr2, 6qf8, and 6v5d) for residues 1-69. There is a slight shift in the center of the AF2 models compared to the NMR structures, though the breadth of the disorder is comparable. 3) Structural overlay of an AF2 model (purple) and NMR model (grey). The AF2 model has PC1 and PC2 values of -0.196 and 0.018, respectively. The NMR structure is 2kox model #23 and has PC1 and PC2 values of 0 and 0.068, respectively. There are slight variations between the two structures, but they are clearly the same fold with no conformational change. 4) Conformation diversity of the ubiquitin AF2 models (purple) and NMR structures (grey).
